# A novel missense variant in the *EML1* gene associated with bilateral ribbon-like subcortical heterotopia leads to ciliary defects

**DOI:** 10.1038/s10038-021-00947-5

**Published:** 2021-07-01

**Authors:** Fenja Markus, Annika Kannengießer, Patricia Näder, Paul Atigbire, Alexander Scholten, Christine Vössing, Eva Bültmann, G. Christoph Korenke, Marta Owczarek-Lipska, John Neidhardt

**Affiliations:** 1 Junior Research Group, Genetics of Childhood Brain Malformations, Faculty VI-School of Medicine and Health Sciences, University of Oldenburg, Oldenburg, Germany; 2 Human Genetics, Faculty VI-School of Medicine and Health Sciences, University of Oldenburg, Oldenburg, Germany; 3 Division of Biochemistry, Biochemistry of signal transduction/neurosensory processes, Faculty VI-School of Medicine and Health Sciences, University of Oldenburg, Oldenburg, Germany; 4 Institute of Diagnostic and Interventional Neuroradiology, Hannover Medical School, Hannover, Germany; 5 Department of Neuropediatrics, University Children’s Hospital, Klinikum Oldenburg, Oldenburg, Germany; 6 Research Center Neurosensory Science, University of Oldenburg, Oldenburg, Germany

**Keywords:** Neurodevelopmental disorders, Neurodevelopmental disorders

## Abstract

Heterotopia is a brain malformation caused by a failed migration of cortical neurons during development. Clinical symptoms of heterotopia vary in severity of intellectual disability and may be associated with epileptic disorders. Abnormal neuronal migration is known to be associated with mutations in the doublecortin gene (*DCX*), the platelet-activating factor acetylhydrolase gene (*PAFAH1B1*), or tubulin alpha-1A gene (*TUBA1A*). Recently, a new gene encoding echinoderm microtubule-associated protein-like 1 (*EML1*) was reported to cause a particular form of subcortical heterotopia, the ribbon-like subcortical heterotopia (RSH). *EML1* mutations are inherited in an autosomal recessive manner. Only six unrelated EML1-associated heterotopia-affected families were reported so far. The EML1 protein is a member of the microtubule-associated proteins family, playing an important role in microtubule assembly and stabilization as well as in mitotic spindle formation in interphase. Herein, we present a novel homozygous missense variant in *EML1* (NM_004434.2: c.692G>A, NP_004425.2: p.Gly231Asp) identified in a male RSH-affected patient. Our clinical and molecular findings confirm the genotype-phenotype associations of *EML1* mutations and RSH. Analyses of patient-derived fibroblasts showed the significantly reduced length of primary cilia. In addition, our results presented, that the mutated EML1 protein did not change binding capacities with tubulin. The data described herein will expand the mutation spectrum of the *EML1* gene and provide further insight into molecular and cellular bases of the pathogenic mechanisms underlying RSH.

## Introduction

A mammalian embryonic telencephalon includes the dorsal telencephalon, which during development generates the neocortex, the ventral subpallium, and the basal ganglia [1]. A dorsal telencephalon can be further divided into three longitudinal pallial regions: medial, lateral, and dorsal. Each of these areas differentiates into different regions of an adult brain, so the medial pallium matures into an archicortex, including a hippocampus; a lateral pallium develops into an olfactory cortex, certain limbic regions, and a partially amygdala; while a dorsal pallium generates a cerebral cortex [1–3]. Cortical development (corticogenesis) is a highly dynamic and specialized process, including neuronal proliferation, differentiation, specification, and cellular migration. Newly generated neuronal cells need to be allocated to final destinations in brain layers and are organized into sensory, motor, and association zones [4, 5]. Abnormalities interfering with corticogenesis often result in a variety of brain malformations [5, 6]. Malformations of cortical development (MCDs) are known to cause intellectual disability of various severity and congenital neurological impairments [6, 7]. They might also be associated with recurrent childhood epilepsy. MCDs can be categorized into three main groups (1–3) according to the developmental stages during which the cortical formation was atypical: (1) reduced proliferation or excess apoptosis (e.g., primary microencephaly, brain overgrowth spectrum, focal cortical dysplasia type IIa/b), (2) abnormal neuronal migration, and (3) abnormal postmigrational development (e.g., dysgyria, focal cortical dysplasia type 1/3, and secondary microencephaly) [8]. The second main group (group 2) can be further classified into five sub-groups: [1] heterotopia, [2] lissencephaly/subcortical band heterotopia, [3] cobblestone, [4] polymicrogyria, and [5] schizencephaly [8]. Heterotopia is arranged into periventricular nodular/laminar heterotopia and subcortical (non-band) heterotopia (e.g., transmantle or ribbon-like heterotopia, sublobar dysplasia, and midline cerebral hamartomas). In all types of heterotopia, neurons left their primary location in the ventricular zone, but failed to achieve their final destinations in the cortex [8–10]. This resulted in a formation of bands of heterotopic gray matter interspersed in the subcortical white matter between the cortex and lateral ventricles [6, 8, 9].

A special form of subcortical heterotopia is called ribbon-like heterotopia and it is characterized by huge, tortuous ribbons of heterotopic gray matter. Usually, the ribbon-like heterotopia symmetrically occurs in both cerebral hemispheres and is associated with corpus callosum agenesis as well as with diffuse polymicrogyria [8, 11].

The first gene associated with heterotopia-affected patients was described in the late 1990s. Variants in the doublecortin gene (*DCX*) were identified in patients with anterior-biased subcortical band heterotopia and/or X-linked lissencephaly [12–15]. Furthermore, sporadic cases with posterior-biased subcortical band heterotopia and lissencephaly were associated with mutations in genes either encoding the platelet-activating factor acetylhydrolase (*PAFAH1B1*) or tubulin alpha-1A (*TUBA1A*) [16–19]. Kielar et al. described an additional gene related to ribbon-like subcortical heterotopia (RSH), a particular form of subcortical heterotopia: The echinoderm microtubule-associated protein-like 1 (*EML1*, OMIM#602033, HP: 0032409). Mutations in *EML1* cause severe heterotopia in mice and bilateral RSH in humans [11]. The EML1 protein is a member of the microtubule-associated proteins family, playing an important role in microtubule assembly and stabilization as well as during interphase mitotic spindle formation [20, 21]. Members of the echinoderm microtubule-associated protein (EMAP) family are evolutionary highly conserved from invertebrates to vertebrates. Until now, this protein family includes six members (EML1–6), which all share the hydrophobic EMAP-like protein (HELP) motif followed by different numbers of WD40 repeats [22, 23]. The core region of these proteins was suggested to fold into a tandem atypical β-propeller (TAPE) domain forming a nearly planar structure with a concave and a convex site [24]. Both β-propellers are linked by the HELP motif. It has been reported that the HELP motif plays an important role in protein folding and in tubulin binding [24–26], while the concave surface of the TAPE domain is responsible for the binding of α/β-tubulin heterodimers [24].

To the best of our knowledge, only eight EML1 mutations, including compound heterozygous mutations (NP_004425.2: p.Arg138* and p.Thr243Ala; NP_004425.2: p.Gly439Asp and p.Gly478Val), homozygous mutations (NP_004425.2: p.Trp225Arg; NP_004425.2: p.Val254Met; NM_001008707: exon 1 deletion arr[hg19] 14q32.2(100,256,118-100,271,376)x0matpat), and nonsense mutation (NP_004425.2: p.Arg523*) were described in six unrelated families with band heterotopia [11, 27–29]. Thus, the EML1-associated band heterotopia -phenotype seems to be a very rare neurological condition.

Herein, we present a novel homozygous missense variant (NM_004434.2: c.692G>A, NP_004425.2: p.Gly231Asp) identified in a consanguineous family affected with RSH. Our clinical and molecular findings expand the understanding of *EML1*-associated pathogenic processes leading to RSH.

## Material and methods

### Human subjects

The young male patient affected with RSH was referred for neurological and neurophysiological examinations to the neuropediatric unit at the Children’s Hospital in Oldenburg (Oldenburg, Germany). Genetic and molecular analyses were performed in the Division of Human Genetics and the Junior Research Group at the University of Oldenburg (Oldenburg, Germany).

All probands were informed about the details and consequences of this study, agreed to participate, and signed the informed written consents form prior to begin of the study. The study adhered to the tenets of the Declaration of Helsinki and it was approved by local ethic committees (Hannover Medical School (MHH) ethics committee [2576-2015], Medizinische Ethik-Kommission University Oldenburg [2018-097]).

### DNA extraction

Peripheral blood samples from the RSH-affected patient and available family members, including the parents and one unaffected brother, were collected in EDTA tubes and sent for molecular analyses. DNA samples were prepared according to manufacturer’s recommendations using Gentra Puregene kit (QIAGEN, Hilden, Germany). Quantity and quality analyses of DNA samples were estimated by Biospectrometer (Eppendorf, Hamburg, Germany) and Qubit^®^ Fluorometer (Thermo Fisher Scientific, Dreieich, Germany).

### Whole exome sequencing (WES) analyses

Genomic DNA samples (total gDNA: 50 ng/sample) from the patient and the unaffected brother (adult) were used for an enzymatic fragmentation/ligation and enrichment of exomic sequences using TruSeq^®^ Rapid Exome Kit (Illumina, San Diego, California, USA) as previously described [30, 31]. Selected sequence variants were verified for an autosomal recessive mode of inheritance, considering homozygous as well as compound heterozygous variants, and also for de novo variants. Sequence variants selected in WES data from the patient were compared with WES data from the unaffected brother. Candidate genes complied with the filtering requirements were verified for known disease-associations in the Human Gene Mutation Database (Professional 2018.3; http://www.hgmd.cf.ac.uk), and in publicly available search databases. Sequencing reads of the patient and the unaffected brother, were also verified for quantity and quality with Integrated Genomics Viewer (http://software.broadinstitute.org/software/igv).

### Primers, PCR amplification, and Sanger sequencing

Forward and reverse primers (fwd_5′-GCCTGTGCCTGTTGAATAAAGC-3′ and rev_5′-GGAGACCACACAGAAGAGCT-3′) located in intronic regions of the human *EML1* gene (NM_004434.2) and flanking exon 7 were designed using Primer Input3 (http://primer3.ut.ee/). They were additionally verified for common single‐nucleotide polymorphisms using SNPCheck (https://secure.ngrl.org.uk/SNPCheck/). In total, 10 ng of each gDNA sample from the patient, and the available healthy relatives were used for PCR amplification with HotFirePol DNA Polymerase (Solis BioDyne, Tartu, Estonia) according to standard protocols. The amplicons were enzymatically purified with ExoI-SAP (New England Biolabs (NEB), Frankfurt, Germany) and bilaterally sequenced using BigDye^®^ Terminator v3.1 Cycle Sequencing Kit on ABI Prism 3130xl Genetic Analyzer (Applied Biosystem, Carlsbad, California, USA). Sanger sequencing data were analyzed with SeqScape (Applied Biosystem) and SnapGene software (GLS Biotech, Chicago, Illinois, USA).

### Multiple species protein alignments, in silico protein domains predictions, and 3D modeling of EML1 protein

Multiple sequence alignments were performed with the ClustalW2 tool (http://www.ebi.ac.uk/Tools/msa/clustalw2/). Amino acid sequences of EML1 proteins were compared between *Homo sapiens* (NP_004425.2), *Mus musculus* (NP_001036800.1), *Rattus norvegicus* (Q4V8C3.2), *Danio rerio* (NP_001180527.1), and *C. elegans* (O45487.1).

Publicly available tools to predict protein domains, such as NCBI Conserved Domain Search (https://www.ncbi.nlm.nih.gov/Structure/cdd/wrpsb.cgi), PROSITE (http://prosite.expasy.org), and SMART (http://smart.embl-heidelberg.de) were used to predict in silico the position of the mutated amino acid residue in the EML1 protein.

Open-source software Swiss-Model (https://swissmodel.expasy.org/) and Jmol (http://jmol.sourceforge.net/) were used to model a three-dimensional (3D) structure of the human EML1 protein (NP_004425.2, encompassing amino acids 175–815) and to visualize the position of the mutated amino acid residue in the patient.

Predictions of possible pathogenicity of missense variants in EML1 were analyzed using MetaDome web server [32].

### Patient-derived fibroblast cell cultures

Patient-derived primary fibroblasts were obtained from skin biopsies from the patient and unrelated controls (C1, C2). The biopsies were prepared as previously described [33, 34]. Skin fibroblast was cultured in Minimal Essential Medium (MEM, Biowest, Nuaillé, France) containing 20% fetal bovine serum (Biowest), l-glutamine (Biowest) as well as antibiotic-antimycotic (Biowest) and were incubated at 37 °C and 5% CO_2_.

### Immunocytochemical (ICC) staining

Skin fibroblasts from the patient, and control cell lines (C1, C2) were initially counted, seeded on coverslips (1.2 × 10^6^ cells per 12 mm coverslip) and incubated overnight in 12-well plates under previously mentioned cell culture conditions. Next day, cells were fixed for 20 min in 4% paraformaldehyde (PFA, Carl Roth, Karlsruhe, Germany) and blocked for 30 min in phosphate-buffered saline (PBS, Chemsolute, Renningen, Germany) containing 0.1% Tween 20 (AppliChem, Darmstadt, Germany) and 5 % bovine serum albumin (BSA, fraction V, Carl Roth). ICC stainings were performed with primary antibodies: EML1 (1:300, EML1 (B-3), sc-390841, Santa Cruz Biotechlology, Dallas, Texas, USA), EML1 (1:600, PA5-21294, Thermo Fischer Scientific), α-Tubulin (1:7500, T9026, Sigma-Aldrich, St. Louis, Missouri, USA) and α-Tubulin (1:300, PA5-16891, Thermo Fischer Scientific) either at room temperature for 2 h or at 4 °C overnight. Skin fibroblasts were afterwards incubated for 2 h at room temperature with secondary antibodies containing 488- or 568-fluorochrome (1:2000, A10037, A10042, A-21202, A-21206, Thermo Fischer Scientific). After incubation cells were trebly washed with PBS, containing 0.2% Tween 20. Coverslips were mounted with DAPI Fluoromount-G (SouthernBiotech, Birmingham, USA). Finally, slides were analyzed with a fluorescence microscope (Olympus, IX83) equipped with DP80 camera (1360 × 1024 pixels). Microscope images were adjusted with ImageJ software (ImageJ, Bethesda, Maryland, USA).

### EML1 protein extraction and western blotting (WB)

Skin fibroblast cells from the patient and control cell lines (C1, C2) were used to generate protein pellets. Cells (1 × 10^6^ cells per pellet) were lysed in 300 µl of radioimmunoprecipitation assay buffer (RIPA, 1% NP40, 5% sodiumdeoxycholate, 0.5% SDS), containing 10% phosphatase inhibitor cocktail 2 and 3 (Sigma-Aldrich), and 40% protease inhibitor complete with EDTA (La Roche, Basel, Switzerland). Cells were incubated for 30 min on ice and centrifuged (6000 g, 10 min, 4 °C). Protein concentrations were estimated with Pierce BCA Protein Assay Kit (Thermo Fischer Scientific). Protein samples (30 µg each) were separated with a 12% polyacrylamide gel and transferred to a polyvinylidene difluoride membrane (pore size: 0.45 µm, Merck Millipore, Burlington, Massachusetts, USA). Membranes were blocked for 1 h with tris-buffered saline, containing 0.1% Tween 20 (TBST) and 5% BSA, followed by overnight incubation with primary antibodies (EML1 (B-3), 1:800, sc-390841, Santa Cruz Biotechnology; EML1, 1:1000, PA5-21294, Thermo Fisher Scientific; GAPDH, 1:1000, MAB374, Merck Millipore; α-Tubulin (1:10,000, T9026, Sigma-Aldrich)) at 4 °C. Afterwards, the membrane was incubated with horse radish peroxidase coated secondary antibodies (1:5,000, NB7539, NB7160, Novus Biologicals, Centennial, Colorado, USA) at room temperature for 1 h and afterwards with enhanced chemiluminescence solution (Thermo Fisher Scientific). All antibodies were initially diluted in TBST, containing 5% BSA. Detection of targeted protein signals was performed with ChemiDoc MP Imaging System (Bio-Rad).

### Measurements of primary cilia length and statistical analyses

In total, 1.6 × 10^5^ human skin fibroblasts were seeded in 12-well culture dishes. Incubation of 24 h was followed by serum starvation for 48 h at 37 °C and 5% CO_2_ (MEM without FCS), and fixation using 4% PFA for 30 min. Blocking and ICC was performed using following antibodies: anti-γ-tubulin (1:500, ab11316, Abcam, Cambridge, UK), anti-detyrosinated α-tubulin (1:1000, D-tubulin, ab3201, Merck Millipore), Alexa Fluor 488, and 568 (1:2000, Life Technologies, Carlsbad, California, USA) as described above.

A Fluorescence microscope (Axiophot, Carl Zeiss, Oberkochen, Germany) equipped with AxioCam camera (Carl Zeiss) and Axiovision software (Carl Zeiss) was used for immunofluorescent images of 240 cilia per cell line (P, C1, C2). The axoneme length of primary cilia was determined with ImageJ software and evaluated with Origin 2017 software (OriginLab, Northampton, Massachusetts, USA). Statistical significance was calculated using SPSS Statistics 24 software (IBM, Armonk, New York, USA) and Mann–Whitney *U* test (*p* ≤ 0.05 = significant).

### Microtubule-association assay

A complete coding sequence of the *EML1* gene (NM_001008707.1) was amplified using cDNA derived from human skin fibroblasts and primers containing restriction sites for XhoI and EcoRI (NEB), with Phusion High-Fidelity DNA Polymerase (Thermo Fisher Scientific; primers: fwd_5′-ACGTTACTCGAGATGGAGGACGGCTTCTCCA-3′, rev_5′-TTCGCGAATTCCTAAATGACGCGCCACTG-3′). After restriction digestions, the insert was ligated into the XhoI/EcoRI cloning site of pIRES2-egfp vector (Takara Clontech, Kusatsu, Shiga Prefecture, Japan) with T4-DNA ligase (Thermo Fisher Scientific).

*Escherichia coli* XL10-Gold ultracompetent cells (Agilent Technologies, Santa Clara, Kalifornien, USA) and NEBstable competent cells (NEB) were transformed by heat shock method and cultivated over night at 37 °C on Luria-Bertani agar plates containing 10% kanamycin for selection. Plasmids containing *EML1*-insert were isolated and purified by Nucleo Spin Plasmid Quick Pure Kit (Macherey and Nagel) according to the manufacturer’s recommendations.

Site-directed mutagenesis reactions were performed using Q5^®^ Site-Directed Mutagenesis Kit (NEB) to generate expression constructs carrying the missense variant (NM_004434.2: c.692G>A, primers: fwd_5′-GGTCTATGGGTACAGGGaTCGAGACTGCCGTAACA-3′, rev_5′-TGTTACGGCAGTCTCGATCCCTGTACCCATAGACC-3′). Plasmids were afterwards verified by sequencing.

Human embryonic kidney cells (HEK-293T) were cultured in Dulbecco’s Modified Eagle Medium (Biowest), supplemented with 8.9% FCS, 0.9% l-glutamine and 0.9% penicillin/streptomycin at 37 °C and 5% CO_2_. In total, 2.5 × 10^6^ HEK-293T cells were seeded in T75 culture dishes. The cells were grown to reach a confluency of 80% for 24 h at 37 °C and 5% CO_2_. Afterwards cells were transiently transfected by the help of polyethylenimine (PEI, Sigma-Aldrich) and plasmid-DNA (10 ng) in a ratio of 4:1 for 24 h. To isolate proteins, the cells were washed once with PBS and scraped off in 300 µl lysis buffer (40 mM HEPES (pH 7.4), 120 mM NaCl, 2 mM EDTA, 0.3% CHAPS), containing phosphatase and protease inhibitors (Cocktail 2 and 3, Sigma-Aldrich; protease inhibitor complete, La Roche). Harvesting of cells was followed by centrifugation (600 g, 3 min, 4 °C). Protein concentrations were determined using Bradford assay (Thermo Fisher Scientific).

Finally, 200 ng protein was incubated by rotating end to end for 1 h at 4 °C with EML1 antibody (2 µg/ml, PA5-21294, Thermo Fischer Scientific). Dynabeads Protein G (Thermo Fisher Scientific; 20 µl) pre-washed with lysis buffer were added and incubated by rotating for 1 h at 4 °C. After triple washing with lysis buffer, elution of bound protein was performed using pH-shift (0.1 M citrate, Carl Roth, pH 2.5). Further on, pH of each eluate was adjusted by adding 1 M Tris/HCl pH 8. The WB analyses were performed with EML1 ((B-3), 1:800, sc-390841, Santa Cruz Biotechnology), GAPDH (1:1000, MAB374, Merck Millipore), and α-Tubulin (1:10,000, T9026, Sigma-Aldrich). A mock control (empty vector), a control for transfection reagent (PEI), and wild-type protein form HEK-293T cells were used as controls.

## Results

**Fig. 1:**
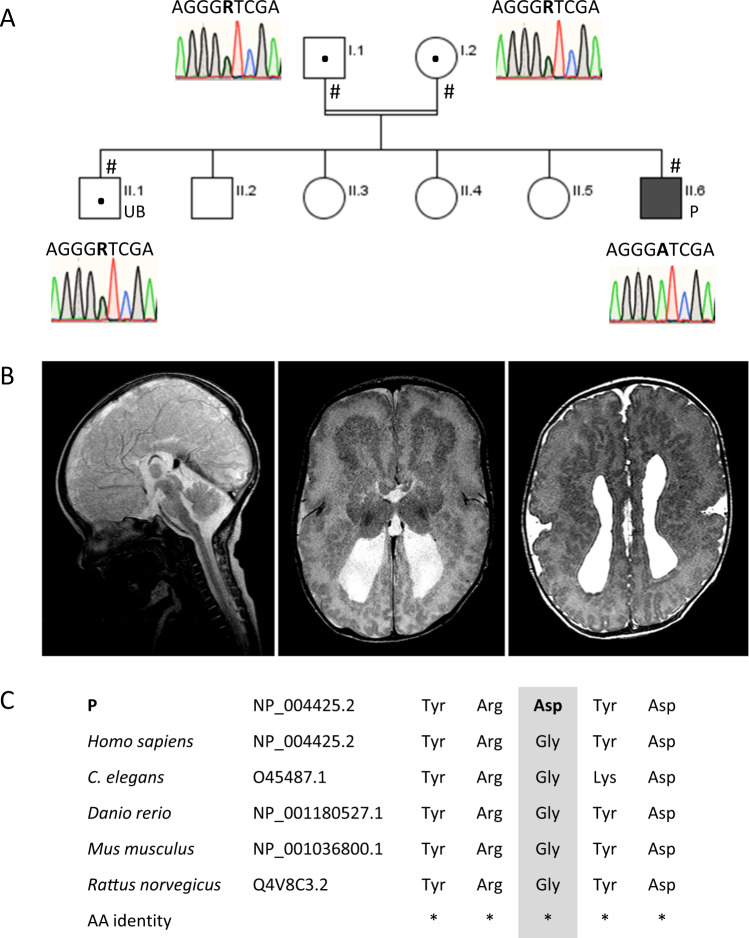
Clinical manifestations of RSH in the patient and familiar co-segregation analyses the novel missense variant in the *EML1* gene. **A** The pedigree of the family showing the RSH-affected family member (P, II.6). The parents are third-grade cousins. Co-segregation analyses of the novel missense variant (NM_001008707.1: c.746G>A) in the *EML1* gene revelated that the healthy parents (I.1 and I.2) and the brother (UB, II.2) were carriers, while the RSH-affected patient (P, II.6) showed both mutated alleles. **B** Sagittal and axial T2 weighted images at age 3 weeks demonstrate complete callosal agenesis, bilateral enlargement of the occipital horns (colpocephaly), hypoplastic basal ganglia, and a complex cortical malformation. The polymicrogyric cortex is thin and irregular in all lobes. Bilaterally an undulating ribbon-like heterotopic band of gray matter is visible in subcortical location partially reaching the periventricular margin. **C** Multiple protein sequence alignment of EML1 proteins showed that the amino acid residue 231 is highly conserved across species (NP_004425.2, gray). Squares—males, circles—females, black solid square—patient affected with RSH, solid white icons—healthy individuals, double line—consanguinity, P—RSH-affected patient, UB—unaffected brother,  #—family members available for further analyzes including Sanger sequencing, AA—amino acid, asterisk indicates highly conserved AA positions

The 11-year-old patient is the sixth child of healthy consanguineous parents (third-degree cousins). Five older siblings (male and female) were healthy (Fig. 1A). The family originated from Lebanon.

In the patient, agenesis of corpus callosum was diagnosed intrauterine by ultrasound in the 28th week of gestation. Delivery was performed by caesarean section at 37th week of gestation due to a macrocephaly (head circumference 39 cm, *z* = 3.0). Other parameters were normal: body weight 3750 g (*z* = 1.5), body length 53 cm (*z* = 1.2), Apgar values 10–10 at 5 and 10 min, and umbilical artery pH 7.37.

Postnatal cranial ultrasonography confirmed the finding of a callosal agenesis with consecutive colpocephaly and additionally reduced gyration pattern and echogenic changes in the thalamus. Ultrasound examinations of the abdomen and heart as well as the ophthalmological examination were unremarkable. MRI scans performed at the age of 3 weeks confirmed a complex brain structure disorder with callosal agenesis, colpocephaly, hypoplastic basal ganglia as well as a complex gyration disorder (Fig. 1B). An irregular, microgyral brain surface with a narrow cortex band showing a “lumpy-bumpy” surface and an overall abnormal gyration pattern was found. The actual cortex is separated by a broad band of unmyelinated white matter from an irregular heterotopic band of gray matter, with a finely plicated run of subcortical, laminar, heterotopic gray matter (double cortex; Fig. 1B). This band partially reaches the ventricular surface in the sense of subependymal heterotopia.

At the age of 8 months, a single febrile seizure with post-convulsive left arm paresis occurred which persisted for 48 h. Epileptic seizures did not develop in the further course. Electroencephalogram examinations did not show any epileptiform activity.

The patient manifested a profound global developmental delay. He learned to walk freely at the age of 4 ½ years, speaks 5–6 single words at the age of 10 years and is only able to understand some simple sentences. He showed a behavioral disorder with restlessness and a short attention span and has a severe sleep disorder with problems to fall asleep and waking up several times during the night. Development of head circumference was accelerating with crossing the percentiles due to the ongoing supratentorial overgrowth resulting in megalencephaly. At the age of 10 years, the head circumference was 60.2 cm (3.9 cm > P97; *z* = 4.9), whereas body length (143 cm, *z* = 0.2) and body weight (30.4 kg, *z* = −0.6) were normal.

We performed extensive metabolic diagnostics of organic acidurias, aminoacidurias, lysosomal and peroxisomal diseases, congenital disorders of glycosylation syndromes, as well as cerebrospinal fluid examinations. These examinations were unremarkable.

Routine cytogenetic chromosome examination and DCX gene sequence analyses (carried out during the first months of life) did not detect pathogenic alterations. Array CGH analyses identified 6 small deletions (chr. 7p22.3, 10q21.3, 12q24.33, 17q15.3, 19p13.3, 19p13.3; see Supplementary Table 1), all of which were also maternally inherited and thus were considered to be familiar non-pathogenic variants. Furthermore, a diagnostic molecular genetic brain malformation panel (50 genes) did not identify sequence alterations associated with a diagnosis of RSH. In order to complement the search for a molecular genetic cause of RSH, the patient was directed to us to perform WES.

### Dual-based WES analysis

All coding regions of the genome were analyzed using WES in blood samples from the RSH-affected patient and the unaffected brother. A multiplex sequencing run resulted in 27.4 Gb raw data, showing Q30-score of 91.9%, cluster density of 232 K/mm^2^, and cluster passing filter of 88.4%. After de-multiplexing and annotation, the sequencing data from the patient showed 110.34 M reads, 92% of 10x targeted coverage, and 79,076 sequence variants (30,822 homozygous and 41,265 heterozygous). Similarly, sequencing data from the unaffected brother revealed 106.51 M of reads, 91.06% of 10x targeted coverage, and 79,694 sequence variants (29,483 homozygous and 42,950 heterozygous). The WES data set was initially used to analyze a panel of 432 genes associated with different types of brain malformations and neurodevelopmental syndromes. In the patient, from 3384 sequence variants only 38 passed the filtering criteria for reliable and potentially pathogenic sequence alterations. All these sequence variants were verified in the unaffected brother and afterwards finally excluded. The analyses were extended to all genes/variants in the WES datasets of both, the patient and unaffected brother. Among the 561 identified sequence variants, a homozygous missense substitution (NM_004434.2: c.692G>A, NP_004425.2: p.Gly231Asp) in the *EML1* gene was identified in the patient. This variant was detected in a heterozygous state in the unaffected brother. The gene was mapped to chromosome 14q32.2 (chr14:99,793,413-99,942,060) and the newly identified *EML1* missense variant located to chr14 at position 99,897,160 (hg38_ncbiRefSeqCurated_NM_004434.2). Allele frequencies of this sequence variant were not reported in either Genome Aggregation Database (GnomAD) or 1000 G databases. In silico predictions suggested that this *EML1-*variant is likely to be disease-causing (SiftRank: 0.91219; MutTasterRank: 0.81).

Additional sequence variants identified by WES analyses were considered unlikely as they were either associated to different clinical phenotypes or did not match the inheritance pattern with the family described herein. Analyses of compound heterozygous variants did not reveal any other plausible candidate gene. In conclusion, the newly identified missense variant in the *EML1* gene provided the only plausible explanation of the RSH phenotype observed in the patient.

### Co-segregation analyses of novel missense variant in the *EML1* gene

Sanger sequencing was performed in the available family members to verify genotype distributions of the missense *EML1*-variant (NM_004434.2: c.692G>A; Fig. 1A). Sequencing results showed that mutated alleles were transmitted to the patient from the mother (I.2) and the father (I.1) confirming co-segregation within the family. The *EML1* sequence variant was identified in a heterozygous state in the unaffected brother (II.1). Co-segregation analyses thus suggested an autosomal recessive mode of inheritance associated with the identified *EML1* sequence alteration c.692G>A (NM_004434.2).

### EML1 protein domains predictions, multiple-species protein sequence alignments, and tolerance analyses

Multiple-species protein sequence alignments of EML1 from *Homo sapiens* (NP_004425.2), *Mus musculus* (NP_001036800.1), *Rattus norvegicus* (Q4V8C3.2), *Danio rerio* (NP_001180527.1), and *C. elegans* (O45487.1) documented a highly conserved glycine at residue 231 of the human EML1 protein reference sequences NP_004425.2 (length 815 amino acids), respectively (Fig. 1C).

**Fig. 2:**
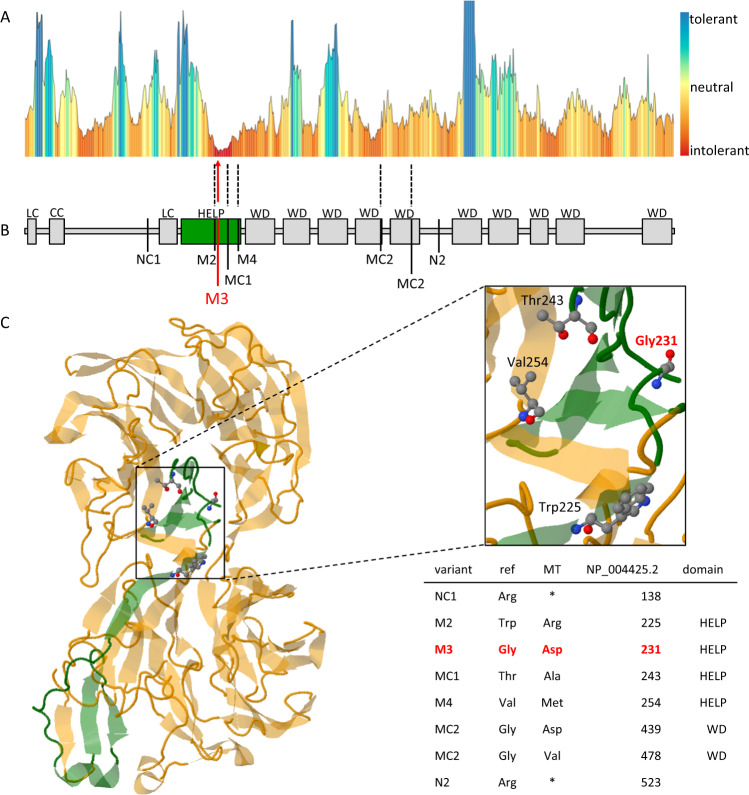
Comparison of known and novel mutations in EML1. **A** Tolerance landscape analyses applying MetaDome showed tolerant (blue), neutral (yellow), and intolerant (red) regions for missense variants. **B** Schematic representation of domains of the EML1 protein located to newly identified mutation (M3: p.Gly231Asp) to the HELP motive (green) of EML1. Previously reported EML1 mutations are shown (NC1: p.Arg138*, MC1: p.Thr243Ala, M2: p.Trp225Arg, N2: p.Arg523*, M4: p.Val254Met, MC2: p.Gly439Asp/p.Gly478Val) [11, 27, 28]. **C** The EML1 protein contains two β-propeller structures building the TAPE domain (yellow) intimately associated with HELP motif (green). The presented EML1 structure includes the protein structure from amino acid 175 to 815 (NP_004425.2). Previously reported mutation sites located in the HELP motif are highlighted as ball-and-stick model in the 3D model and in the zoomed-in area. LC low complexity region, CC coiled coil region, HELP hydrophobic EMAP-like protein motif (green), WD WD40 repeats, N nonsense, M missense, C compound heterozygous mutations, 1–4—patient’s number

In silico predictions of the EML1 protein domains based on the human EML1 protein reference sequence NP_004425.2 revealed that the mutated glycine residue (amino acid 231) locates to the HELP motif (Pfam PF03451, Fig. 2). In the same protein motif, three other EML1 mutations have previously been detected [11, 28] (Fig. 2B). The N-terminal HELP motif is the integral part of the TAPE domain, which is characteristic for EMAPs.

Prediction of genetic tolerance of the novel missense variant in the *EML1* gene (NM_004434.2: c.692G>A) using MetaDome web server revealed a highly intolerant impact at amino acid residue 231 (NP_004425.2: p.Gly231Asp; Fig. 2A). Similarly to our findings, the previously reported mutations in the EML1 HELP motif (NP_004425.2: p.Trp225Arg, p.Thr243Ala, p.Val254Met) [11, 28] were predicted to be intolerant or highly intolerant.

### Localization of EML1 protein in human skin fibroblasts

**Fig. 3:**
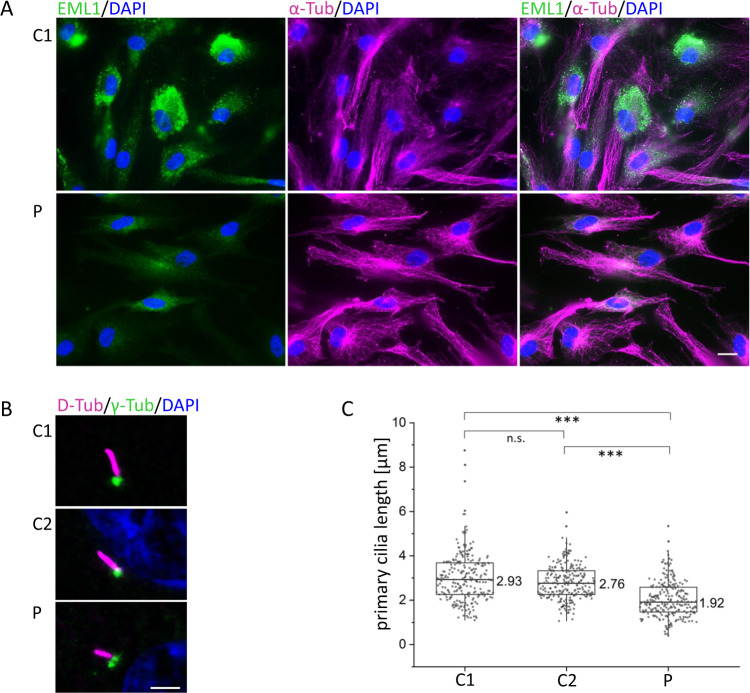
Immunocytochemical detection of EML1 in patient-derived fibroblasts and measurements of primary cilia length. **A** Immunocytochemical detection of EML1 in skin fibroblasts derived from the RSH-affected patient (P) and controls (C1; C2 similar results, data not shown) was performed with antibodies against EML1 (green, PA5-21294,), α-tubulin (magenta, T9026), and DAPI (blue) in four independent replicates. The scale bar corresponds to 20 µm. **B** Cilia axonemes and basal bodies were stained (in patient-derived and control fibroblasts) using acetylated D-tubulin (magenta) and y-tubulin (green), respectively. A scale bar corresponds to 3 µm. **C** Cilia lengths were measured in skin fibroblast from the RSH-affected patient (P) and control cell lines (C1, C2) by measuring the length of D-tubulin signals. At least 240 cilia were measured per cell line. We found no significant difference (*p* > 0.05) in the cilia length between the controls, while a highly significant reduction of cilia length (*p* ≤ 0.001) was observed in the patient when compared to the control cell lines. Symbols: α-Tub α tubulin, γ-Tub γ tubulin, D-Tub D-tubulin, n.s. not significant, Asterisk indicates highly significant

We analyzed the impact of the novel homozygous missense alteration (NP_004425.2: p.Gly231Asp) in patient-derived cells from the RSH-affected family member and compared the results to unrelated control cells. We performed immunocytochemistry to detect possible differences in tubulin association, miss-localization, or cytosolic distributions of EML1. The patient-derived skin fibroblasts were incubated with a polyclonal rabbit EML1 antibody directed against the C-terminus of the EML1 protein. We found equal cytosolic distribution, but observed reduced EML1 signals in the patients' fibroblasts compared to controls (Fig. 3A). The EML1 accumulated in perinuclear regions. Of note, these analyses did not show conclusive evidence for an association of EML1 with microtubular structures, neither in controls nor in fibroblast derived from the RSH-affected patient (data not shown).

### Primary cilia show reduced length in patient-derived cell lines

Skin fibroblasts derived from the patient were analyzed for ciliary properties and compared to controls. Cilia were induced by serum starvation of the cultured fibroblasts. Gamma and D-tubulin antibodies were applied to detect the ciliary basal body and axoneme, respectively. The length of the axoneme was measured and showed a highly significant reduction of the primary cilia lengths in comparison to unrelated control cell lines. The controls showed primary cilia of an average length of 2.93 and 2.76 µm, while the patient-derived cells presented with 1.92 µm long cilia (Fig. 3B, C). Our measurements suggested that the novel homozygous missense variant (NM_004434.2: c.692G>A, NP_004425.2: p.Gly231Asp) in the *EML1* gene cause significantly shorter cilia in the RSH-affected patient described herein.

### EML1 protein expression and association with tubulin

**Fig. 4:**
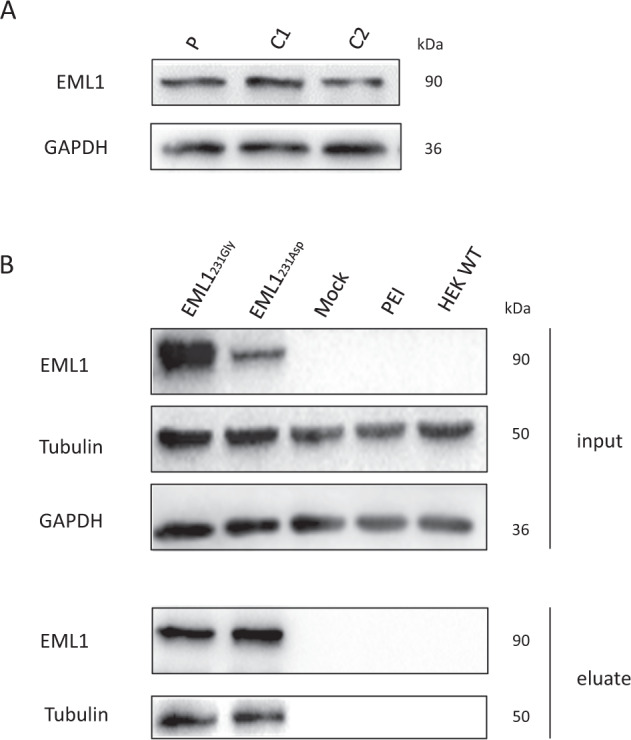
Western blot and co-immune precipitation analyses of the EML1 protein. **A** WB analyses of EML1 protein isolated from fibroblasts derived from the RSH-affected patient (P) and unrelated controls (C1, C2; independent replicates: 3). GAPDH served as a loading control. **B** Overexpression of wild-type EML1_231Gly_ and mutated EML1_231Asp_ in HEK-293T. A mock control (empty vector), a control for transfection reagent (PEI), and wild-type protein form HEK-293T cells were used as controls. Using whole-cell lysate EML1 protein was precipitated by EML1 antibody (PA5-21294) and WB analyses were performed with EML1 (sc-390841) and α-tubulin (T9026) antibodies. P—RSH-affected patient, C1-2—control 1-2, HEK WT—HEK wild type, Mock—empty vector, PEI—polyethylenimine (transfection reagent), EML1_231Gly_—overexpressed EML1 reference protein, EML1_231Asp_—overexpressed EML1 mutated protein, kDa—kilo Dalton

To verify that ICC signal intensities for EML1 were reduced in the patient-derived cell lines, we performed western blot analyses. The western blots of whole-cell protein lysates isolated from skin fibroblasts did not suggest that the EML1 protein expression was reduced in the patient. The size of EML1 was unaltered between the RSH-affected patient and unrelated controls (C1, C2). The EML1 protein was detected at ~90 kDa (Fig. 4A).

Expression constructs for reference EML1 (EML1_231Gly_) and mutated EML1 (EML1_231Asp_) were generated and transfected in HEK-293T cells. Again, we found stronger expression of EML1_231Gly_ compared to EML1_231Asp_ (Fig. 4B), suggesting that the sequence alteration described herein interferes with the expression, folding or stability of EML1.

The cell cycle-dependent EML1 was shown to be associated with microtubules in the interphase [11]. It has been speculated that mutations located in the highly conserved HELP motif may interfere with the depolymerized/polymerized microtubule association of EML1 [11]. To verify this assumption, we performed immuno-precipitation assay. Our analyses suggested that both, unaltered and mutated EML1 bind to depolymerized tubulin with similar affinities (Fig. 4B).

## Discussion

We presented a novel missense variant (NM_004434.2: c.692G>A, NP_004425.2: p.Gly231Asp) in the *EML1* gene identified in an RSH-affected patient.

It has been reported that missense mutations located in the TAPE domain cause incorrect protein domain folding in EMAP family members and it is not responsible for microtubule binding as previously suggested [23] (Fig. 2C). The newly identified homozygous missense variant also located to the TAPE domain and thus, may disrupt the folding of this domain. It has been previously speculated that the TAPE domain is required for a specific cytosolic localization of EML1 and the binding to polymerized microtubules [23]. Supportively, defects of the microtubule cytoskeleton are frequently involved in neuronal migration disorders [35]. Although the function of the microtubule-associated *EML1* remains unclear, it has been mentioned in the literature that this may be important for distribution and function of progenitors in the early developing cortex [11, 36]. Observations in mouse models have shown that heterotopia was formed due to an abnormal radial glial cell distribution, leading to a permanent arrest of the neurons of the upper layer in the neocortical white matter [11, 36]. Furthermore, it has been reported that EML1 is important for the proper orientation of the cleavage plane of neuronal progenitors during cell division, but the neuronal localization of EML1 is poorly understood [37]. It is also speculated that the negatively charged surface of microtubules may influence electrostatically interaction between microtubules and the N-terminal region of EML1, containing the TAPE domain [23]. We observed altered distributions and weaker signal intensities in perinuclear regions of EML1 protein in the fibroblasts derived from the RSH-affected patient in comparison to the unrelated controls. Moreover, we did not observe a diminished association between tubulin and the mutated EML1_231Asp_ protein. Our results mirrored previously reported findings, showing a filamentous signal pattern of the wild-type EML1 and diffuse and weaker signals of the mutated EML1. These results suggested that EML1 mutations do not directly interfere with tubulin binding [11]. Interestingly, our ICC stainings revealed quite decreased expression levels of the mutated EML1 in the fibroblast from the RSH-affected patient. We did not confirm these expression changes with WB analyses.

Moreover, fibroblasts derived from the RSH-affected patient showed shortened cilia. These observations suggest that the new *EML1*-variant may lead to altered ciliogenesis or reduced stability of primary cilia. To clarify the pathogenicity and the impact of variants in the *EML1* gene on cilia, wild typic reference EML1 could be overexpressed in patient-derived fibroblasts to show a rescue of cilia. Our results are in line with recently published data, showing that mutations in the *EML1* gene changes formation and/or assembly of cilia in RSH-affected patients' fibroblasts as it has also been shown in human induced pluripotent stem cell-derived cortical progenitors, and mouse apical radial glias [38]. Uzquiano et al. found shorted primary cilia from EML1-affected patient-derived fibroblasts after 96 h serum starvation. Herein, a significant decrease of primary cilia length in fibroblasts of the RSH-affected patient was already observed after 48 h of serum starvation. These findings indicate a robust effect of EML1 mutations on ciliary properties, likely involving ciliary stability or maintenance. Nevertheless, the exact mechanism underlying the shortened cilia in *EML1* patient-derived cells remains unclear. Notably, ciliogenesis is a highly complex and sensitive cellular process that may be impaired/altered by many endo- and exogenous factors [39].

Furthermore, it has been suggested that different mutations in the *EML1* gene may result in the altered severity of RSH phenotypes [11, 27, 28]. About 6 years ago, a first study described pathogenic variants in the *EML1* gene and associated these variants with the RSH phenotype [11]. Two unrelated families were reported. Since that time, only five additional EML1 mutations ((1) NM_004434.2: c.1567C>T, (2) Arr[hg19] 14q32.2 (100,256,118-100,271,376) x0matpat, (3) NM_004434.2: c.760G>A, (4) c.1316G>A, and (5) c.1433G>T) were reported [27–29]. The c.1567C>T (NM_004434.2) variant was detected in a consanguineous Saudi Arabian family with band heterotopia, congenital hydrocephalus, profound global developmental delay, and intractable epilepsy [27]. Another patient (Arr[hg19] 14q32.2 (100,256,118-100,271,376) x0matpat) from non-related Libaneese parents was reported in 2019 with agenesis of the corpus callosum, severe global developmental delay and ribbon-like heterotopia in the subcortical region [28]. The same study presented a Pakistani patient (NM_004434.2c.760G>A) from consanguineous parents suffering from severe developmental delay, congenital hydrocephalus, epilepsy, and RSH [28]. All the recently reported RSH-affected patients manifested similar clinical symptoms of RSH with different degree of global developmental delay [11, 27, 28]. The patient described herein, also showed typical clinical phenotype of RSH. Therefore, we speculate that the molecular localizations of the various *EML1*-associated mutations in the all RSH-affected cases may not have a direct influence on severity of the clinical features.

Abnormal neuronal migration is also associated with mutations in the *DCX*, *PAFAH1B1*, and *TUBA1A* genes. The *DCX* gene is responsible for organization and stability of microtubule and interacts with the *PAFAH1B1* gene. This interaction is essential for a correct microtubule function in the developing cortex [40]. Heterotopia or lissencephaly-affected patients carrying mutations in these three genes manifest analogous clinical symptoms to the RSH-affected individuals with *EML1*-associated mutations. We also verified the *DCX*, *PAFAH1B1*, and *TUBA1A* genes in WES data from the RSH-affected patient and we did not find any additional sequence variants.

A double cortex has also been reported in *in-vivo* rodent models resembling the human RSH phenotype [11, 41, 42]. Similar to the Eml1-knockout tish-rats, the HeCo and *Eml1*^*tvrm360*^ mice with truncated and non-functional Eml1 protein display the bilateral heterotopic cortex [11, 41, 42].

The few cases reported worldwide suggest a very rare occurance of the RSH condition caused by mutations in the *EML1* gene. To better understand the biological pathway underlying the pathomechanisms of EML1-mediated incorrect neuronal migration, followed by axonal outgrowth and brain wiring, leading to RSH, it will be essential to analyze additional RSH-affected patients molecularly and clinically with the aim to further correlate genotypic and phenotypic findings.

In this paper, we report an additional family with an RSH-affected family member suffering from a novel homozygous missense variant in the *EML1* gene. Our results extend the spectrum of *EML1* mutations and contribute to the understanding of the biomedical aspects involved in the development of RSH.

## Supplementary information

Supplementary table 1
