# Supplementary material for: A novel missense variant in the EML1 gene associated with bilateral ribbon-like subcortical heterotopia leads to ciliary defects
Source: J Hum Genet. 2021 Jul 1;66(12):1159–67. doi: 10.1038/s10038-021-00947-5 (PMC8612930; doi:10.1038/s10038-021-00947-5)
Supplement: Supplementary file 1 — Supplementary table 1 [file 10038_2021_947_MOESM1_ESM.docx]

**Supplementary data**

**Supplementary tab 1: Deletions detected by array CGH analyses.**

| deletion [kb] | chr | Position  [Mb] | Genes | *Disease associations |
| --- | --- | --- | --- | --- |
| 17 | 7p22.3 | 1.434.711-1.451.853 | *MICALL2* | Joubert Syndrome 1 Retinitis Pigmentosa 44 |
| 88.5 | 10q21.3 | 67.307.911-67.396.359 | *CTNNA3* | Arrhythmogenic Right Ventricular Dysplasia, Familial, 13 Familial Isolated Arrhythmogenic Ventricular Dysplasia, Biventricular Form |
| 18 | 12q24.33 | 130.887.114-130.904.869 | n/a |  |
| 10 | 17q25.3 | 77.602.490-77.612.303 | n/a |  |
| 14 | 19p13.3 | 3.433.545-3.447.478 | *NFIC* | Mechanical Ectropion Scrotum Neoplasm |
| 11 | 19p13.3 | 6.700.929-6.712.580 | *C3* | Complement Component 3 Deficiency Hemolytic Uremic Syndrome, Atypical 5 |

n/a – not available, * disease association according to GeneCards – The Human Gene Database (https://www.genecards.org/)
